# Rosiglitazone Ameliorates Adverse Effects of High-Fat Diet in Largemouth Bass (*Micropterus salmoides*): Modulation of Lipid Metabolism, Antioxidant Capacity, Inflammatory Response, and Gut Microbiota

**DOI:** 10.3390/antiox14101230

**Published:** 2025-10-14

**Authors:** Ying Yan, Yanjie Tang, Xiting Chen, Xuan Chen, Muzi Zhang, Dexiang Feng, Ming Li

**Affiliations:** 1 School of Marine Sciences, Ningbo University, Ningbo 315211, China; 2401130144@nbu.edu.cn (Y.Y.); 2301130097@nbu.edu.cn (Y.T.); 2411130076@nbu.edu.cn (X.C.); 2411130006@nbu.edu.cn (X.C.); 2 College of Animal Science, Guizhou University, Guiyang 550025, China; mzzhang3@gzu.edu.cn; 3 School of Fisheries, Xinyang Agriculture and Forestry University, Xinyang 464000, China; dxfeng@xyafu.edu.cn

**Keywords:** *Micropterus salmoides*, rosiglitazone, high-fat diet, Nrf2, oxidative stress, environmental tolerance

## Abstract

High-fat (HF) diets are widely used in aquaculture to reduce feed costs, but they often lead to hepatic steatosis, oxidative stress, and reduced environmental tolerance in carnivorous fish. This study evaluated whether dietary rosiglitazone (RO; 10 mg·kg^−1^) alleviates HF (18% fat) diet-induced metabolic dysfunctions in juvenile largemouth bass (*Micropterus salmoides*). Fish were fed a control diet (10% fat), an HF diet (18% fat), or an HF + RO diet for 8 weeks. RO supplementation reversed HF-induced dyslipidemia by lowering plasma triglyceride (TG) and total cholesterol (T-CHO) while elevating high-density lipoprotein cholesterol (HDL-c), and it reduced intraperitoneal fat and whole-body lipid (*p* < 0.05). RO also mitigated hepatic vacuolization and decreased plasma alanine aminotransferase (ALT) (*p* < 0.05) and aspartate aminotransferase (AST) (*p* > 0.05) activities. Antioxidant capacity was enhanced by RO, as indicated by increased glutathione (GSH), catalase (CAT), and total antioxidant capacity (T-AOC), together with reduced malondialdehyde (MDA), and accompanied by upregulation of *nrf2*, downstream antioxidant genes, and downregulation of *keap1* (*p* < 0.05). Moreover, RO suppressed HF-induced endoplasmic reticulum (ER) stress (*grp78*, *eif2α*, *chop*) and pro-inflammatory genes (*tnfα*, *il-1β*, *nf-κb*), while upregulating *il-10* (*p* < 0.05). Gut microbiota analysis showed RO-mediated enrichment of Firmicutes and short-chain fatty acid-producing genera (*Faecalibaculum*, *Dubosiella*). Importantly, RO significantly reduced mortality during a 96 h acute ammonia challenge (*p* < 0.05). Collectively, these results demonstrate that dietary rosiglitazone mitigates HF diet-induced hepatic oxidative stress and metabolic dysregulation through Nrf2 activation, anti-inflammatory effects, and microbiota modulation, providing a potential strategy to enhance HF feed utilization and environmental stress resilience in carnivorous fish. Further studies on dose optimization and residue safety are warranted.

## 1. Introduction

Reducing fishmeal use is a major focus in aquatic nutrition studies [1]. Primary strategies to address this include utilizing alternative protein sources and incorporating high-fat (HF) diets [2,3]. As fat is an essential energy substrate for fish and exerts a protein-sparing effect [4], increasing dietary lipid levels can lower the dependence on protein and help reduce feed expenses [5]. Nevertheless, excessive or long-term intake of HF diets may lead to metabolic imbalances, excessive lipid deposition, suppression of immune function, endoplasmic reticulum (ER) stress, oxidative damage, and higher mortality rates in aquaculture species [6]. Prior investigations have revealed that HF feeding adversely affects the growth performance of largemouth bass (*Micropterus salmoides*), grass carp (*Ctenopharyngodon idellus*), and black seabream (*Acanthopagrus schlegelii*) [7,8,9,10]. The most direct impact of an HF diet on aquatic species is hepatic lipid deposition, which promotes fatty liver disease [11]. As the central site for lipid metabolism in fish, the liver plays a crucial role in regulating lipid balance and maintaining overall metabolic homeostasis [12]. Hepatocytes are able to absorb circulating free fatty acids and synthesize lipids de novo, subsequently storing triglycerides (TG) in lipid droplets. However, excessive dietary lipid intake disrupts this metabolic balance, leading to systemic dysregulation of lipid metabolism. Furthermore, pathological accumulation of lipids within hepatic tissue induces oxidative stress and inflammatory responses [13,14].

The gut microbiota plays a vital role in regulating fish metabolism, nutrient utilization, and health. It contributes to nutrient digestion and absorption, produces bioactive metabolites such as short-chain fatty acids (SCFAs), and modulates immune and inflammatory responses [15,16,17,18,19]. Consequently, alterations in gut microbial composition can strongly affect lipid metabolism, oxidative balance, and disease resistance. Dietary strategies that modulate gut microbiota have been shown to enhance growth, feed efficiency, and stress tolerance in aquaculture species, underscoring the importance of maintaining intestinal homeostasis [20].

**Figure 1:**
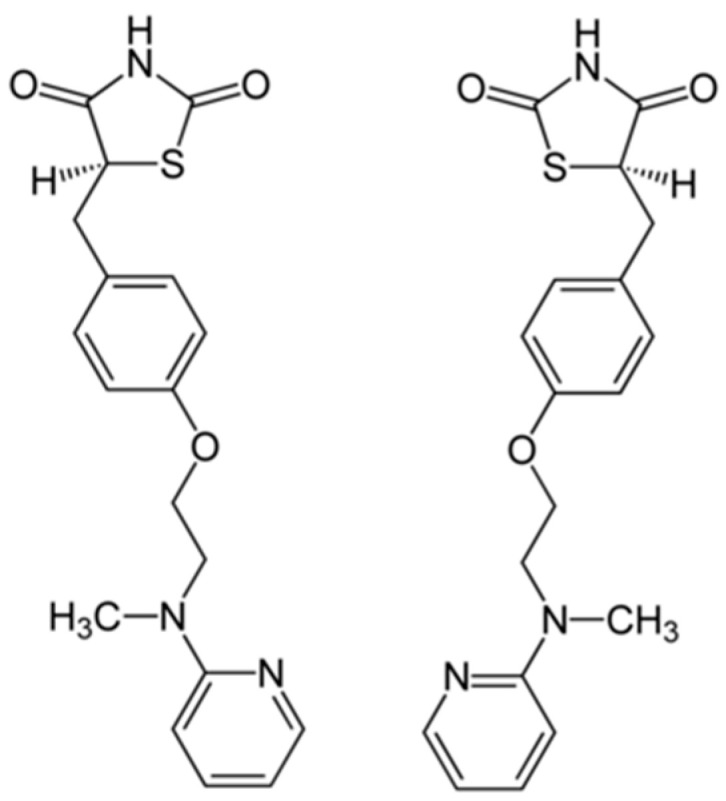
Chemical structure of rosiglitazone (C_18_H_19_N_3_O_3_S), a thiazolidinedione derivative commonly used as a PPARγ agonist.

Rosiglitazone (RO), a synthetic ligand of the nuclear receptor peroxisome proliferator-activated receptor gamma (PPARγ) [21,22], functions in the regulation of glucose (GLU) and lipid metabolism at the physiological level [23,24], and its chemical structure is shown in Figure 1. Clinically employed against diabetes and insulin resistance [25,26], RO also enhances antioxidant capacity and exerts anti-inflammatory effects in mammals [27,28,29,30]. For instance, it has been shown to attenuate oxidative damage and restore redox balance in models of hepatic and cardiac injury, as well as suppress lipopolysaccharide (LPS)-induced inflammatory responses in macrophages through modulation of oxidative stress–related enzymes and signaling pathways [31,32,33].

The established functional properties of RO in mammalian systems—particularly its modulation of lipid metabolism, antioxidant capacity, and anti-inflammatory responses—are prompting emerging interest in its potential as a dietary supplement for farmed fish. In mammals, these effects are largely mediated through activation of the PPARγ pathway, which regulates adipogenesis, lipid oxidation, and inflammatory signaling. Since HF diets in fish induce similar pathophysiological challenges—such as hepatic lipid accumulation, oxidative stress, and inflammatory damage—RO may exert comparable protective effects by activating PPARγ-dependent and antioxidant pathways in aquatic species. Wei et al. [34] confirmed that dietary supplementation with RO enhanced the tolerance to HF diets in grass carp. Also, Guan et al. [35] supported the idea that incorporating RO into a high-starch dietary formulation exerts a beneficial effect on the growth performance of GIFT tilapia (*Oreochromis niloticus*). To date, no studies have investigated the effects of dietary RO on carnivorous fish, particularly largemouth bass. The largemouth bass, a carnivorous species native to North America, has experienced steadily increasing production in China since its introduction in the 1980s and now represents an economically important food fish [36,37]. HF diets are commonly employed in the aquaculture of largemouth bass [38]. However, HF diet feeding induces prevalent metabolic disturbances, including lipid deposition, dyslipidemia, physiological stress, inflammation, and apoptosis in largemouth bass [7,38,39]. Therefore, this study was designed to investigate whether dietary RO could mitigate HF diet-induced metabolic dysfunction, oxidative stress, and inflammation in juvenile largemouth bass.

## 2. Materials and Methods

### 2.1. Experimental Diets and Feeding Management

In this trial, three diets were formulated: a basal control (Con) diet, a high-fat diet (HF), and a rosiglitazone-supplemented diet (RO). The HF diet was supplemented with an additional 4% fish oil and 4% soybean oil compared to the Con diet, while the crude protein content remained consistent between the two groups. The RO treatment diet was formulated by adding 0.001% rosiglitazone (equivalent to 10 mg/kg) to the HF formula. The dosage of RO was determined based on recent studies in grass carp [34] and GIFT tilapia [35]. Feed pellets measuring 2 mm and 4 mm were fabricated using a screw-type pelletizer (CD2-1TS, Guangzhou Huagong Optoelectronic Technology Co., Ltd., Guangzhou, China). The pellets subsequently underwent high-temperature conditioning, followed by drying at 60 °C. The dried samples were maintained at −20 °C to preserve their stability until further use. Detailed dietary composition and nutritional analysis are presented in Table 1.

We sourced juvenile largemouth bass from Xinyang Shuihong Aquaculture Co., Ltd. (Xinyang, China). Following acquisition, the fish were acclimated for two weeks in a recirculating aquaculture system (RAS) while being fed the Con diet twice daily, followed by a one-day fasting period. A total of 270 healthy juvenile fish of similar size (mean initial weight: 3.26 ± 0.02 g) were randomly assigned to nine 300-L tanks, with 30 individuals per tank. The three dietary treatments were distributed in triplicate according to a completely randomized design. Fish were hand-fed twice daily (07:00–08:00 and 17:00–18:00) over an 8-week period. During the feeding trial, any uneaten feed was siphoned out 30 min after each feeding, dried at 60 °C to a constant weight, and subtracted from the total feed offered to calculate daily feed intake (FI = total feed supplied − uneaten feed collected by siphoning). The RAS was run continuously, providing 24 h water circulation and constant aeration to keep dissolved oxygen levels above 6.0 mg/L. Water quality parameters were monitored every 48 h, with partial water exchanges performed to maintain optimal conditions. During the 8-week feeding experiment, the environmental conditions were controlled as follows: water temperature 28–29 °C, pH between 7.0 and 8.0, ammonia levels kept below 0.3 mg/L, photoperiod set at 12L:12D, and natural light intensity.

### 2.2. Sample Collection

Upon termination, all fish underwent a 24 h fasting period prior to euthanasia via MS-222 anesthesia. Sampling procedures commenced with enumeration and weighing of all fish per tank to calculate final body weight (FBW), weight gain rate (WGR), specific growth rate (SGR), survival rate (SR), feed conversion ratio (FCR), protein efficiency ratio (PER), FI, and feeding rate (FR). Subsequently, three fish per tank (a total of 9 per treatment group) were randomly selected for biometric measurements (body weight and length), followed by blood collection via caudal venipuncture for serum biochemical assays (*n* = 3 per group), liver sampling for antioxidant enzyme activity measurements (*n* = 6 per group), and liver sampling for histology (*n* = 3 per group). Necropsy included documentation of visceral mass, liver weight, and intraperitoneal adipose tissue weight for determination of hepatosomatic index (HSI), viscerosomatic index (VSI), condition factor (CF), and intraperitoneal fat ratio (IPF). Liver tissues were perfused with sterile phosphate-buffered saline (PBS), with bile duct-adjacent sections fixed in 4% paraformaldehyde (PFA). The remaining liver and intestinal samples were rapidly frozen in liquid nitrogen and kept at −80 °C for later biochemical analyses, gene expression analyses (*n* = 6 per group), and 16S rRNA sequencing for microbiota analysis (*n* = 4 pooled samples per group). At the same time, six fish were randomly selected from each treatment group to determine whole-body proximate composition.

### 2.3. Ammonia Challenge Test

Following sampling, 15 fish of uniform size were selected from each remaining tank and exposed to 50 mg/L total ammonia nitrogen (TAN) at pH 8.0 ± 0.2 and 28 °C for 96 h. TAN concentrations were maintained through daily measurements and adjustment with ammonium chloride. Feeding was suspended throughout the stress challenge period. Mortality was monitored every 12 h, and surviving fish were not used for subsequent analyses. The 96 h cumulative mortality rate (CMR) was determined using the formula: CMR (%) = 100 × (number of dead fish under ammonia challenge/15).

### 2.4. Analytical Procedures

Feed and whole-body proximate compositions, including moisture, crude protein, crude lipid, and ash, were assessed according to established protocols [40,41]. In brief, moisture was determined by drying samples at 105 °C until a constant weight was reached; ash was analyzed by combusting the dried material in a muffle furnace at 550 °C for 5 h; crude protein content was calculated using the Kjeldahl method (N × 6.25); and crude lipid was quantified by Soxhlet extraction with petroleum ether.

Plasma biochemical parameters, including total protein (TP, A045-2-1), GLU (A154-1-1), TG (A110-1-1), total cholesterol (T-CHO, A111-1-1), high-density lipoprotein cholesterol (HDL-c, A112-1-1), low-density lipoprotein cholesterol (LDL-c, A113-1-1), alanine aminotransferase (ALT, C009-1-1), and aspartate aminotransferase (AST, C010-1-1), were measured with commercial assay kits provided by Nanjing Jiancheng Bioengineering Institute (Nanjing, China). Similarly, hepatic antioxidant parameters, including malondialdehyde (MDA, A003-1-2), total antioxidant capacity (T-AOC, A015-2-1), glutathione (GSH, A006-2-1), catalase (CAT, A007-1-1), and superoxide dismutase (SOD, A001-1-2), were assayed using standardized commercial kits by Nanjing Jiancheng Bioengineering Institute (Nanjing, China).

### 2.5. Hepatic Gene Expression Profiles

Total RNA was extracted from liver tissues using TRIzol™ reagent (Takara Bio, Beijing, China). RNA integrity was verified by spectrophotometry (SuperMiro, SM-100, Shanghai Xituo Scientific Instruments Co., Ltd., Shanghai, China), with OD_A230/A280_ between 1.8 and 2.0 deemed acceptable for downstream processing. cDNA synthesis and quantitative PCR (qPCR) were performed following our established laboratory protocol [42]. qPCR was carried out using the following cycling parameters: 95 °C for 30 s, followed by 40 cycles of 95 °C for 5 s and 60 °C for 30 s. Primers were designed against the target sequences using NCBI Primer-BLAST and commercially synthesized by Ningbo Leyi Gene Technology Co., Ltd. (Ningbo, China). The detailed primer information is provided in Table 2. Relative gene expression was calculated using the 2^−ΔΔCt^ method with *β-actin* and *ef1α* as the endogenous control.

### 2.6. Tissue Sectioning

Liver tissues were fixed, dehydrated using a graded ethanol series, cleared with xylene, and embedded in paraffin. Sections of 5 μm thickness were cut using a rotary microtome (YD-202A, Jinhua Yidi Medical Equipment Co., Ltd., Jinhua, China) and subjected to hematoxylin and eosin (H&E) staining using reagents purchased from Nanjing Jiancheng Bioengineering Institute (Nanjing, China; D006-1-1). The stained sections were then observed and photographed under a Nikon TS100 light microscope (Nikon, Tokyo, Japan) at 200× magnification (scale bar = 40 μm). Histological assessment was qualitative, focusing primarily on the degree of hepatocellular vacuolization and structural integrity of hepatic tissue.

### 2.7. 16S rRNA Sequencing and Analysis

Shanghai Yuanxin Biotechnology Co., Ltd. (Shanghai, China) was responsible for extracting, purifying, and verifying total intestinal DNA, as well as designing primers specific to the V4–V5 regions. Genomic DNA of intestinal microbiota was extracted using a commercial kit (Omega Bio-Tek, Norcross, GA, USA). The concentration and purity of the extracted DNA were determined with a NanoDrop 2000 UV-Vis spectrophotometer (Thermo Scientific, Wilmington, NC, USA). The hypervariable V3–V4 region of the bacterial 16S rRNA gene was amplified with primer pair 338F (5′-ACTCCTACGGGAGGCAGCAG-3′) and 806R (5′-GGACTACHVGGGTWTCTAAT-3′) using an ABI GeneAmp^®^ 9700 PCR thermocycler (ABI, Foster City, CA, USA). Raw paired-end sequencing reads were first quality-filtered using FASTP (https://github.com/OpenGene/fastp, accessed on 2 April 2025; version 0.20.0), and the filtered reads were then merged with FLASH (https://ccb.jhu.edu/software/FLASH/, accessed on 4 April 2025; version 1.2.7). Sequence analysis was performed using UPARSE (http://drive5.com/uparse/, accessed on 8 April 2025; version 7.1), with sequences showing ≥97% similarity clustered into operational taxonomic units (OTUs). Chimeric sequences were identified and removed using UCHIME. Taxonomic classification of each OTU representative sequence was conducted using the RDP Classifier (https://sourceforge.net/projects/rdp-classifier/, accessed on 11 April 2025; version 2.2). α-diversity indices (ACE, Chao1, Shannon, and Simpson, all calculated based on the Kruskal-Wallis test), linear discriminant analysis (LEfSe), and heat-tree analysis (based on the hierarchical taxonomic structure, with microbial community differences between groups compared using the Wilcoxon rank-sum test) were all performed with the online tool MicrobiomeAnalyst (https://www.microbiomeanalyst.ca/, accessed on 15 April 2025).

### 2.8. Statistical Analysis

Data normality was assessed using the Shapiro-Wilk test, and homogeneity of variance was evaluated with Levene’s test. Parametric data were analyzed by one-way ANOVA followed by Tukey’s post hoc test, whereas nonparametric data were examined using the Kruskal-Wallis test. Continuous data are presented as mean ± standard error of the mean (SEM), and differences were considered statistically significant at *p* < 0.05. All statistical analyses were performed using SPSS version 26.0 (IBM Corp., Armonk, NY, USA).

## 3. Results

### 3.1. Growth Performance and Body Indexes

Growth performance results are shown in Table 3. After the 8-week feeding trial, the FBW in all treatment groups increased nearly tenfold compared to initial values. Compared to the Con, the HF group showed significantly decreased FBW, FI, WGR, and SGR (*p* < 0.05). Supplementation with RO in the HF diet increased these metrics (WGR and SGR) compared to the HF group, although the improvement did not reach statistical significance (*p* > 0.05). Similarly, no significant intergroup differences were detected in SR, FR, FCR, and PER (*p* > 0.05).

The HF group exhibited significantly higher HSI, VSI, and IPF compared to the Con (*p* < 0.05). Concomitantly, the CF was higher in the HF, albeit statistically insignificant (*p* > 0.05). RO treatment obviously reduced VSI compared with the HF (*p* < 0.05). Notably, these parameters (VSI, CF, and IPF) in the RO reverted to levels comparable to the Con, with no significant differences observed (*p* > 0.05).

### 3.2. Whole-Body Composition

**Figure 2:**
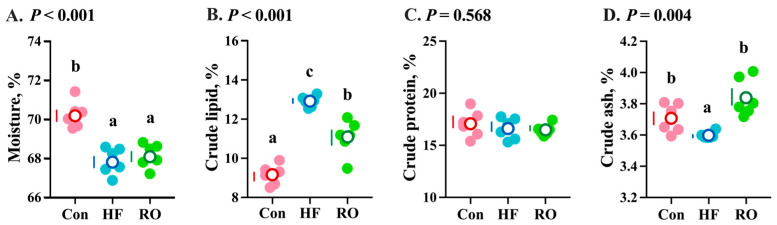
Whole-body composition of largemouth bass under dietary treatment (*n* = 6). (**A**) Moisture. (**B**) Crude lipid. (**C**) Crude protein. (**D**) Crude ash. Data were expressed as the mean ± SEM (*n* = 6). Significant differences among groups (*p* < 0.05) were indicated by different letters on the petal diagrams.

Compared to the Con, the HF treatment group exhibited marked reductions in whole-body moisture and crude ash contents (Figure 2A,D; *p* < 0.05), concomitant with a significant elevation in crude lipid content (Figure 2B; *p* < 0.05), with no alteration in crude protein content (Figure 2C; *p* > 0.05). Supplementation of RO in the HF diet significantly decreased crude lipid content and increased crude ash content (*p* < 0.05) compared to the HF group.

### 3.3. Plasma Lipid Profiles

**Figure 3:**
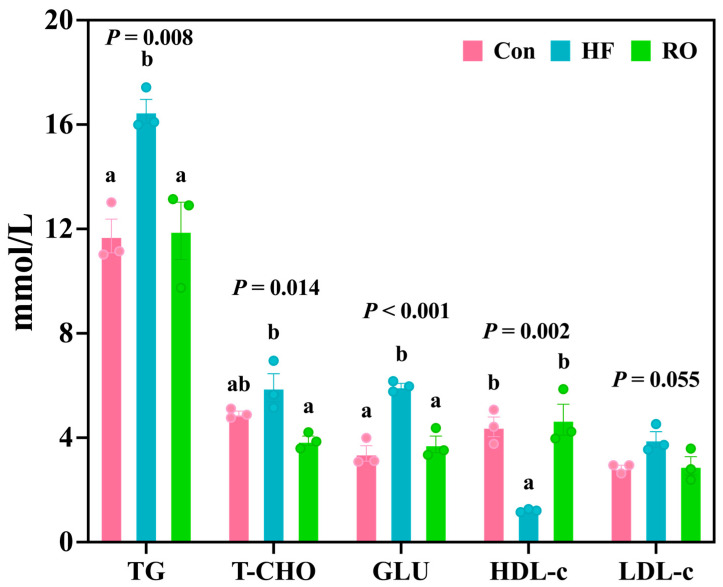
Blood biochemical parameters of largemouth bass under dietary treatment (*n* = 3). TG = triglyceride; T-CHO = total cholesterol; GLU = glucose; HDL-c = high-density lipoprotein cholesterol; LDL-c = low-density lipoprotein cholesterol. All data presented as means ± SEM (*n* = 3). Significant differences among groups (*p* < 0.05) were indicated by different letters on the bar charts.

The HF group demonstrated pronounced hyperlipidemia (Figure 3), with significantly elevated TG and GLU levels (vs. Con, *p* < 0.05) and reduced HDL-c (*p* < 0.05). In addition, LDL-c levels in the HF group were slightly higher than those in the Con group (*p* > 0.05). RO effectively attenuated these alterations, significantly lowering TG, T-CHO, and GLU, while elevating HDL-c compared with the HF (*p* < 0.05). Moreover, the Con and RO treatment groups showed no significant differences in TG, T-CHO, GLU, HDL-c, and LDL-c levels (*p* > 0.05).

### 3.4. Lipid Metabolism-Related Genes

**Figure 4:**
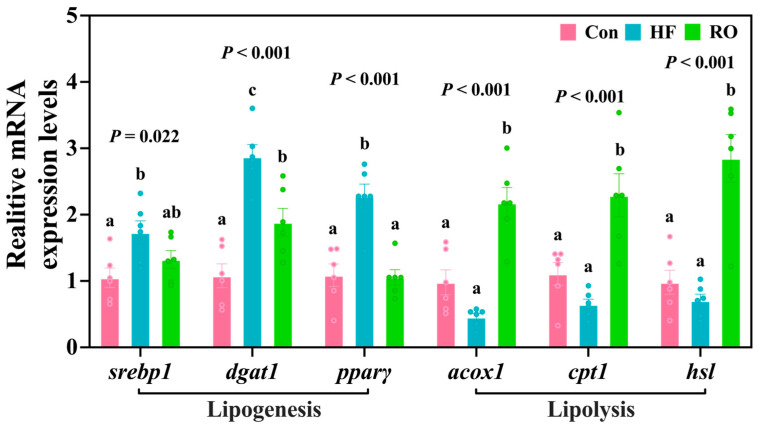
Liver lipid metabolism-related mRNA expression in largemouth bass under dietary treatment (*n* = 6). *srebp1* = sterol regulatory element binding protein 1; *dgat1* = diacylglycerol oacyltransferase 1; *pparγ* = peroxisome proliferator-activated receptor γ; *acox1* = acyl-CoA oxidase 1; *cpt1* = carnitine palmitoyltransferase1; *hsl* = hormone-sensitive lipase. All data presented as means ± SEM (*n* = 6). Significant differences among groups (*p* < 0.05) were indicated by different letters on the bar charts.

The results of lipid metabolism-related mRNA expression are displayed in Figure 4. Compared to the Con, the HF exhibited significant upregulation of *srebp1*, *dgat1*, and *pparγ* mRNA expression (*p* < 0.05). Supplementation with RO significantly downregulated *dgat1* and *pparγ* mRNA compared to HF (*p* < 0.05). Notably, *srebp1* and *pparγ* mRNA expression levels in RO were comparable to those in the Con (*p* > 0.05). HF downregulated the *acox1*, *cpt1*, and *hsl* mRNA expression vs. Con (*p* > 0.05). RO supplementation obviously upregulated all three genes (*acox1*, *cpt1*, or *hsl*) compared to both Con and HF (*p* < 0.05).

### 3.5. Liver Histology and Plasma Aminotransferase Levels

**Figure 5:**
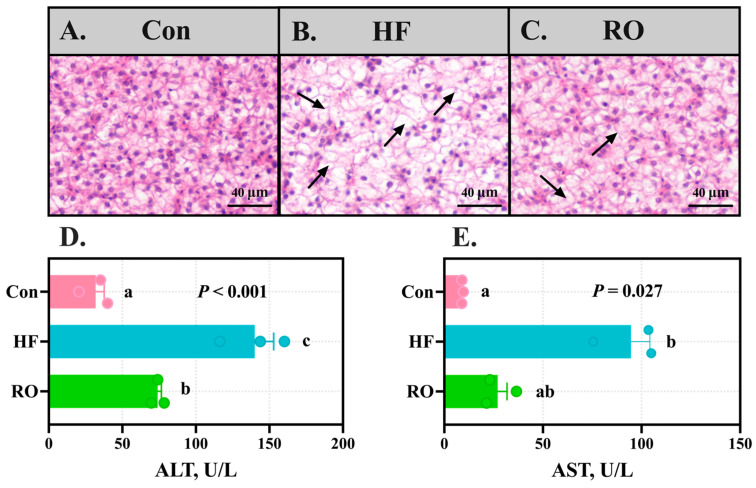
Liver structure and plasma aminotransferase levels of largemouth bass fed various dietary treatments (*n* = 3). (**A**–**C**) Hematoxylin and eosin staining of the liver in (**A**) Con, (**B**) HF, and (**C**) RO group. Black arrows indicate hepatocellular vacuolation. Scale bar = 40 µm (200×). (**D**) ALT levels in plasma. (**E**) AST levels in plasma. All data presented as means ± SEM (*n* = 3). ALT = alanine aminotransferase. AST = aspartate aminotransferase. Significant differences among groups (*p* < 0.05) were indicated by different letters on the bar charts.

Hepatic histology revealed marked vacuolization in HF (Figure 5A–C). While the RO group exhibited improved parenchymal architecture compared to HF, residual vacuolization persisted relative to Con. Concomitantly, ALT and AST levels in plasma were obviously elevated in HF versus Con (Figure 5D,E; *p* < 0.05). RO significantly ameliorated these elevations (*p* < 0.05), with AST in RO showing no obvious disparity from Con (*p* > 0.05).

### 3.6. Hepatic Antioxidant Parameters

**Figure 6:**
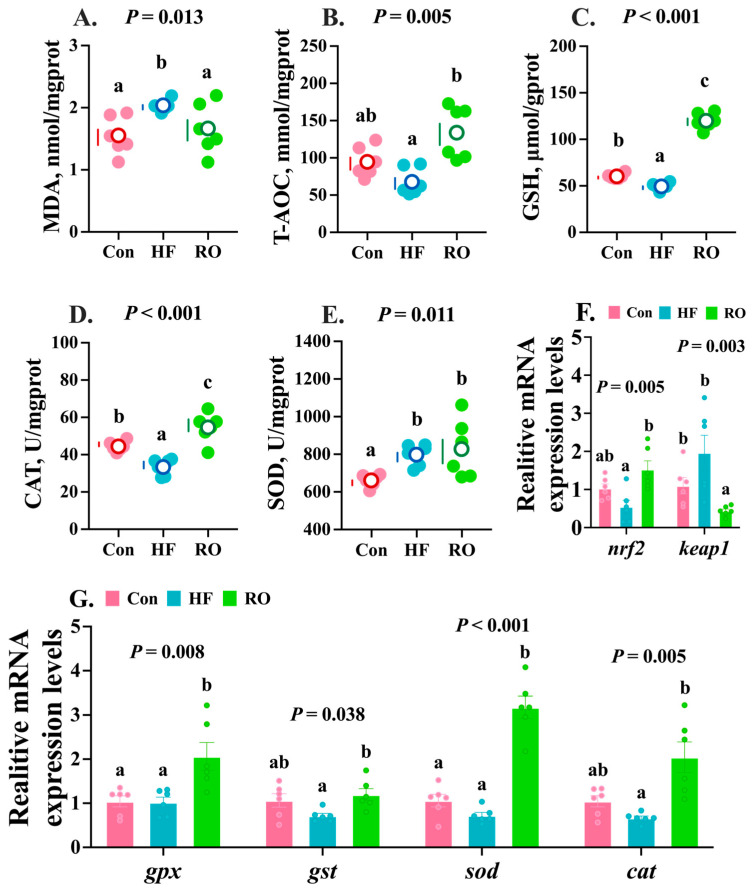
Hepatic antioxidant parameters of largemouth bass fed various dietary treatments (*n* = 6). (**A**–**E**) Antioxidant enzymes (*n* = 6). (**F**,**G**) Relative mRNA expression levels of *nrf2*-*keap1* signaling pathway genes (*n* = 6). MDA = malondialdehyde; T-AOC = total antioxidant capacity; GSH = glutathione; CAT = catalase; SOD= superoxide dismutase; *nrf2* = nuclear factor erythroid 2-related factor 2; *keap1* = kelch-like ECH-associated protein 1. All data presented as means ± SEM (*n* = 6). Significant differences among groups (*p* < 0.05) were indicated by different letters on the petal diagrams and bar charts.

The HF treatment group exhibited the highest hepatic MDA content, significantly exceeding that of the Con and RO groups (Figure 6A; *p* < 0.05). Interestingly, the RO group showed slightly higher MDA content compared to Con (Figure 6A; *p* > 0.05). In contrast, the HF group displayed the lowest hepatic T-AOC activity (Figure 6B), which was significantly lower than that of the RO group (*p* < 0.05), while no significant difference was observed between the Con and RO groups (*p* > 0.05). The HF group showed significantly diminished hepatic GSH (Figure 6C) content and CAT (Figure 6D) activity vs. Con (*p* < 0.05). Notably, the RO group achieved maximal values for these parameters, demonstrating significant elevations over both the Con and HF groups (*p* < 0.05). Compared to Con, SOD activity was significantly increased in other treatment groups (Figure 6E; *p* < 0.05).

The HF group exhibited the lowest relative mRNA expression of *nrf2*, which was notably lower than that in RO (Figure 6F; *p* < 0.05). Concurrently, *keap1* mRNA expression was highest in the HF group and obviously elevated vs. RO (Figure 6F; *p* < 0.05). Relative to the Con group, RO significantly downregulated *keap1* expression (*p* < 0.05), but not on *nrf2* (*p* > 0.05). Meanwhile, the mRNA expression levels of *gpx*, *gst*, *sod*, and *cat* in the HF group showed no significant differences compared with the Con group (Figure 6G; *p* > 0.05). Notably, RO supplementation significantly upregulated the expression of these antioxidant genes relative to the HF group (Figure 6G; *p* < 0.05). Furthermore, the expression levels of *gst* and *sod* were markedly higher in the RO group than in the Con group (Figure 6G; *p* < 0.05).

### 3.7. Inflammation-Related mRNA Expression

**Figure 7:**
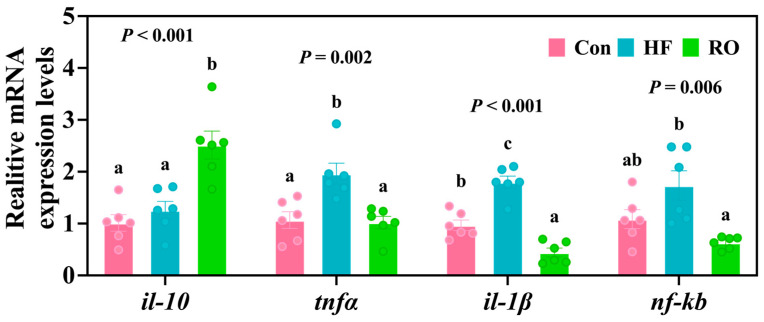
Liver inflammation-related mRNA expression of largemouth bass fed various dietary treatments (*n* = 6). *il-10* = interleukin-10; *tnfα* = tumor necrosis factor α; *il-1β* = interleukin 1β; *nf-κb* = nuclear factor kappa-B. All data presented as means ± SEM (*n* = 6). Significant differences among groups (*p* < 0.05) were indicated by different letters on the bar charts.

The HF group exhibited the obviously upregulated expression of *tnfα* and the *il-1β* gene vs. Con (Figure 7; *p* < 0.05). Furthermore, relative to the HF group, RO treatment significantly upregulated *il-10* mRNA expression while downregulating *tnfα* mRNA, *il-1β* mRNA, and *nf-κb* mRNA expression (*p* < 0.05). Of note, the RO group also demonstrated significantly elevated *il-10* mRNA levels accompanied by significant downregulation of *il-1β* gene when compared to Con (*p* < 0.05).

### 3.8. ER Stress-Related mRNA Expression

**Figure 8:**
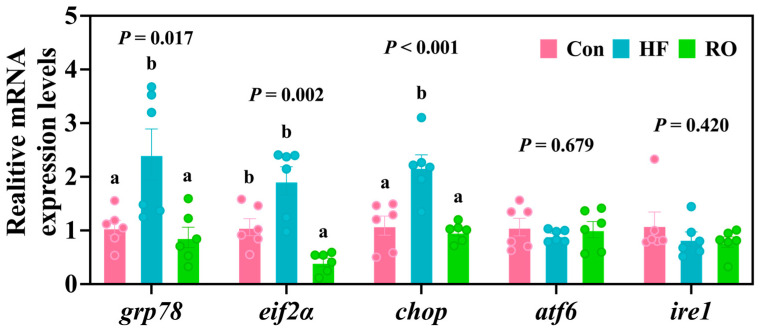
Liver ER stress-related mRNA expression in largemouth bass under dietary treatment (*n* = 6). *grp78* = glucose regulated protein 78; *eif2α* = eukaryotic initiation factor-2α; *chop* = C/EBP-homologous protein; *atf6* = activating transcription factor 6; *ire1* = inositol-requiring enzyme 1. All data presented as means ± SEM (*n* = 6). Significant differences among groups (*p* < 0.05) were indicated by different letters on the bar charts.

Compared with the Con group, the HF group exhibited a marked elevation in *grp78* mRNA and *chop* mRNA (Figure 8; *p* < 0.05). RO supplementation markedly downregulated the expression of all three genes (*grp78*, *eif2α*, and *chop*) relative to the HF group (*p* < 0.05). Interestingly, *eif2α* mRNA levels were also significantly lower in the RO group than in the Con group (*p* < 0.05). Meanwhile, no significant variations were detected in the expression of *atf6* mRNA and *ire1* mRNA among the experimental groups.

### 3.9. Gut Microbiota

**Figure 9:**
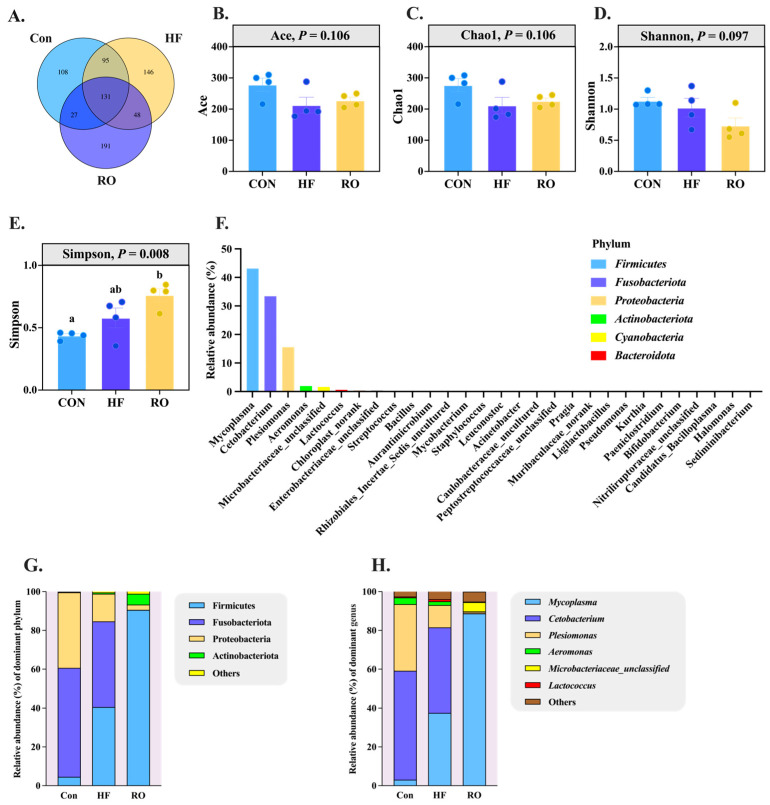
Disparity in gut microbiota of largemouth bass fed various dietary treatments (*n* = 4). (**A**) Venn analysis. (**B**) Ace species richness index. (**C**) Chao1 species richness index. (**D**) Shannon diversity index. (**E**) Simpson diversity index. (**F**) Microbial community barplot at phylum level. (**G**) Gut microbiota at phylum level. (**H**) Gut microbiota at genus level. Significant differences among groups (*p* < 0.05) were indicated by different letters on the bar charts.

**Figure 10:**
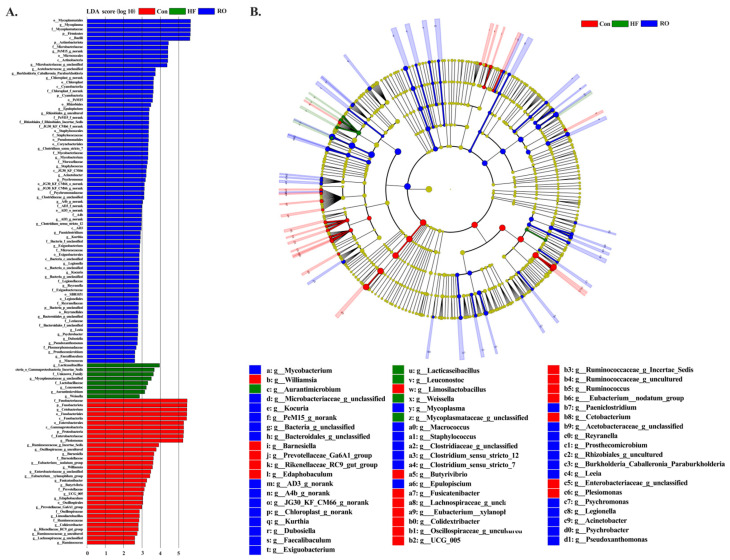
Linear discriminant analysis (LDA) and taxonomic cladogram illustrating differentially abundant taxa across groups (*n* = 4). (**A**) Histogram of LDA scores for discriminative features. The vertical axis represents taxonomic units showing significant differences, and the horizontal axis corresponds to the LDA score (log10). (**B**) Cladogram depicting phylogenetic distribution and abundance of key taxa. Node size reflects relative abundance; red, green, and blue nodes indicate taxa enriched in the Con, HF, and RO groups, respectively.

The intestinal microbiota composition of largemouth bass was analyzed across the three dietary treatments (Figure 9 and Figure 10). Venn diagram analysis showed 361, 420, and 397 OTUs in the Con, HF, and RO groups, respectively, indicating minor differences in overall microbial richness (Figure 9A). Alpha diversity indices (ace, chao1, and shannon) did not differ significantly among groups (*p* > 0.05), while the Simpson index was significantly higher in the RO group than in the Con group (*p* < 0.05) (Figure 9B–E), suggesting slightly increased community evenness with RO supplementation. At the phylum level, Firmicutes, Fusobacteriota, Proteobacteria, Actinobacteriota, and Cyanobacteria were dominant in all groups (Figure 9F). Compared with the Con and HF groups, the RO group showed a significantly greater abundance of Firmicutes and Actinobacteriota (*p* < 0.05), while Fusobacteriota and Proteobacteria were significantly reduced (*p* < 0.05). The HF group, relative to Con, also exhibited an increase in Firmicutes and a decrease in Proteobacteria (Figure 9G; *p* < 0.05). At the genus level, RO-fed fish showed higher relative abundances of *Mycoplasma* and *Microbacteriaceae_unclassified* but lower abundances of *Cetobacterium* and *Plesiomonas* compared with both Con and HF groups (*p* < 0.05). Within the HF group, *Mycoplasma* increased and *Plesiomonas* decreased significantly relative to Con (Figure 9H; *p* < 0.05).

LEfSe analysis (LDA > 2.0, *p* < 0.05) identified a total of 119 differentially enriched genera (Figure 10), among which *Limosilactobacillus*, *Ruminococcaceae*, *Oscillospiraceae*, *Barnesiella*, and *Butyrivibrio* were enriched in the Con group, while *Lacticaseibacillus*, *Gammaproteobacteria*, *Leuconostoc*, *Aurantimicrobium*, and *Weissella* were enriched in the HF group, while *Dubosiella, Faecalibaculum, Kurthia*, and *Exiguobacterium* were enriched in the RO group.

To facilitate the interpretation of microbial changes, the main taxa that increased or decreased under each dietary treatment are summarized in Table 4.

### 3.10. Ammonia Stress

**Figure 11:**
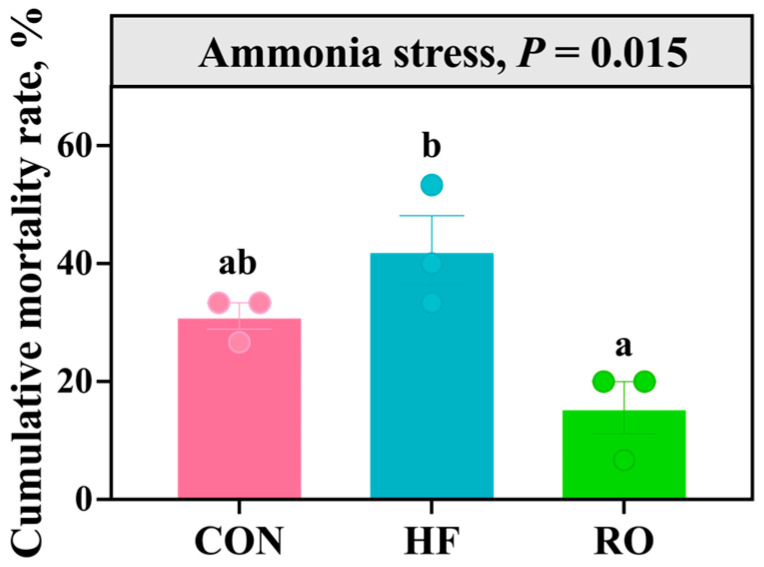
The cumulative mortality rate of largemouth bass under 96 h ammonia stress under dietary treatment (*n* = 3). All data presented as means ± SEM (*n* = 3). Significant differences among groups (*p* < 0.05) were indicated by different letters on the bar charts.

Figure 11 illustrates the cumulative mortality rate of largemouth bass over 96 h of ammonia stress under different dietary treatments. While the HF diet increased mortality compared to the Con group (*p* > 0.05), its mortality rate was elevated significantly relative to the RO group (*p* < 0.05). Critically, the RO group exhibited reduced mortality compared to the Con group (*p* > 0.05), counteracting HF-induced effects.

## 4. Discussion

### 4.1. Growth and Lipid Metabolism

HF diets are considered a viable nutritional strategy for intensive aquaculture. However, excessive dietary lipid levels often promote lipid deposition, subsequently triggering inflammatory responses, ER stress, and oxidative stress damage [43,44,45]. These issues ultimately result in reduced growth performance and increased mortality. The HF (18% fat) diet significantly suppressed growth performance indicators (FBW, WGR, SGR) in fish relative to the Con (10% fat) diet, consistent with studies on black seabream [9], yellow catfish (*Pelteobagrus fulvidraco*) [46], and largemouth bass [38]. Notably, despite the adverse effects of the HF diet, dietary RO supplementation provided a slight improvement in growth. This growth-enhancing effect of RO in HF diets aligns with previous observations in GIFT tilapia [35].

The most direct adverse effect of HF diets on farmed animals is induced lipid deposition [47]. Our results demonstrate that HF significantly increased morphometric indices, including the HSI and IPF, while markedly elevating whole-body lipid content. These changes were further accompanied by significantly higher plasma levels of TG and GLU. These findings are consistent with previous studies [48,49,50]. The aberrant lipid accumulation observed likely stems from a disruption in the balance between lipid uptake/synthesis and catabolism [51]. In this study, HF significantly upregulated hepatic lipogenesis-associated genes (*srebp1*, *dgat1*, and *pparγ*) while downregulating lipid degradation genes (*acox1*, *cpt1*, and *hsl*), indicating coordinated metabolic reprogramming toward fat accumulation. Excitingly, RO treatment reduced the IPF and whole-fish fat content, as well as plasma TG and T-CHO levels, while significantly increasing plasma HDL-c levels and the expression of lipid degradation genes (*acox1*, *cpt1*, and *hsl*). Concerning T-CHO, these findings suggest that RO may exert a direct anti-cholesterolemic effect, beyond merely counteracting high-fat–induced metabolic stress. Furthermore, an intriguing phenomenon was noted in the present study: despite the well-established role of RO as a PPARγ agonist, *pparγ* activation was not detected in the liver tissue of largemouth bass. Consistent findings were reported in grass carp, where dietary RO supplementation in an HF diet failed to induce *pparγ* activation in hepatic and muscle tissues, while such activation was observed in adipose tissue [34]. We speculate that this tissue-specific response reflects differences in receptor distribution, post-translational regulation, or tissue-specific cofactors that affect PPARγ activation. These observations have practical implications for aquaculture, suggesting that PPARγ agonists may act differently across tissues and species, and that targeted strategies could help optimize lipid metabolism and reduce HF diet-induced metabolic stress. Further studies are warranted to elucidate the molecular basis of this selective activation.

### 4.2. Hepatic Health and Oxidative Stress

Dietary HF regimens in aquatic species commonly induce lipid peroxidation and oxidative stress, leading to hepatic injury [14,52,53]. In this study, largemouth bass fed HF diets showed pronounced hepatocellular vacuolation and elevated plasma ALT and AST levels, indicative of hepatic dysfunction [40,41]. Crucially, RO supplementation attenuated histopathological vacuolation and normalized transaminase concentrations by regulating lipid metabolism to reduce hepatic lipid deposition and inhibiting lipid peroxidation. Further analysis revealed that RO significantly decreased hepatic MDA levels-a key marker of lipid peroxidation and liver health [54,55,56] and enhanced antioxidant capacity, increasing GSH content and CAT activity. Research on RO’s effects on antioxidant status in aquatic species is limited. In CD-1 mice, RO pretreatment mitigated reductions in hepatic glutathione peroxidase (GPX), glutathione reductase (GR), and glutathione S-transferase (GST) activities and prevented GSH depletion during acetaminophen-induced liver injury [57]. Similarly, RO ameliorated CCl_4_-induced alterations in murine serum markers, reversing SOD, CAT, and GSH depletion while reducing ALT, AST, and MDA elevations [31]. RO has also been shown to upregulate Nrf2, a key transcriptional regulator of antioxidative responses [58]. Hepatic steatotic cells experience oxidative stress with impaired metabolism and excessive reactive oxygen species (ROS) generation [59]. Under normal conditions, Nrf2 is bound to and inhibited by its cytoplasmic repressor Keap1 [60]. During oxidative stress, Nrf2 dissociates from Keap1, translocates to the nucleus, and activates transcription of antioxidant response element (ARE)-driven cytoprotective genes [61]. Prolonged oxidative stress may deplete Nrf2 due to excessive activation, impairing antioxidant capacity [62]. Consistent with these observations, our results indicated that RO treatment significantly upregulated hepatic *nrf2* mRNA expression while concurrently downregulating *keap1* mRNA levels.

### 4.3. ER Stress and Inflammation

Due to the close relationship between oxidative stress and ER stress, and given that lipids are synthesized in the ER, HF diets can also induce ER stress [63,64]. Excessive lipid intake can lead to ER stress, and conversely, ER stress itself can also disrupt lipid metabolism. Jia et al. [44] confirmed that a HF diet (12% fat) compared to a control diet (6% fat) resulted in elevated protein expression of GRP78 and EIF2α, as well as upregulated gene expressions of *ire1*, *grp78*, *xbp1s*, and *chop*, confirming the induction of ER stress in tilapia. Additionally, a series of studies confirmed that a 19% HF diet induced ER stress in black seabream, as evidenced by the elevated expressions of *ire1*, *xbp1*, *grp78*, *perk*, and *atf6* compared to the control group fed 12% fat [48,65,66].

The ER contains three transmembrane receptors that continuously monitor its status [67]. Under normal conditions, these sensors bind the ER chaperone GRP78 and remain inactive [64]. ER stress occurs when misfolded or unfolded proteins accumulate, releasing GRP78 and activating the sensors, which then bind unfolded proteins and trigger downstream signaling to restore protein homeostasis [68]. Additionally, ER stress activates *eif2α*, coordinating global translation inhibition with selective transcription of stress-adaptive genes to help cells manage protein folding stress [69,70]. In the present study, RO intervention significantly reduced the relative mRNA expression levels of *grp78*, *eif2α*, and *chop* in the liver, indicating that RO can effectively alleviate hepatic ER stress induced by HF in largemouth bass. Notably, *eif2α* expression in the RO group was even lower than that in the control group, suggesting that RO may actively suppress basal ER stress signaling rather than merely restoring it to normal levels. This strong downregulation of ER stress markers highlights the potential of RO to maintain ER and metabolic homeostasis.

Given that ER stress and inflammation are tightly interconnected in metabolic disorders, the inhibition of ER stress by RO likely contributes to its anti-inflammatory effects. Consistently, multiple studies have demonstrated that RO exerts potent anti-inflammatory activity [71,72]. RO inhibited hepatic inflammation during the pathogenesis of non-alcoholic steatohepatitis [73,74]. In line with these reports, RO treatment in the present study markedly reduced the mRNA expression of pro-inflammatory mediators (*nf-κb*, *il*-1β, *tnf-α*), while concurrently upregulating the anti-inflammatory gene *il-10*.

### 4.4. Intestinal Microbial Community

Extensive research has shown that fish intestines harbor diverse symbiotic microorganisms, which are crucial for maintaining intestinal development and function, metabolism, immune responses, disease resistance, and overall health [15,16,17,18,19]. The widespread use of HF diets frequently induces gut microbiota dysbiosis. It has been well-established that HF dietary intake induces intestinal microbiota dysbiosis in zebrafish (*Danio rerio*), a phenomenon particularly characterized by a marked increase in the relative abundance of the phylum *Bacteroidetes* [75]. In yellow catfish, HF dietary intake markedly alters the intestinal microbial community structure, leading to a reduction in the abundances of *Proteobacteria* and *Bacteroidetes* alongside an enrichment of *Firmicutes*, a shift that subsequently impairs growth performance [76]. In the present study, the core microbial structure within the intestinal microbiota of largemouth bass demonstrated remarkable stability; even under varying dietary conditions, *Proteobacteria*, *Fusobacteriota*, and *Firmicutes* consistently persisted as the three dominant phyla-a finding consistent with previously reported ecological profiles in this species [77,78,79]. However, following the addition of an HF diet, the relative abundances of these three dominant phyla all underwent significant changes, despite their persistence as the primary dominant phyla in the gut. Previous studies have indicated that an increased relative abundance of Firmicutes in the gut can reduce intestinal inflammation [80]. In the present study, RO administration resulted in a significantly greater relative abundance of Firmicutes compared to both the Con and HF groups, indicating that RO may ameliorate inflammatory responses through modulation of the gut microbial community. Results from LEfSe indicated that RO supplementation significantly enhanced the relative abundance of specific beneficial bacterial genera, notably *Faecalibaculum* and *Dubosiella*. Prior research has demonstrated that *Faecalibaculum* and *Dubosiella*, which generate metabolites like SCFAs, possess notable anti-inflammatory properties [81,82,83].

### 4.5. Ammonia Stress Tolerance

The long-term HF dietary regimen has been reported to increase susceptibility to mass mortality when fish encounter environmental stressors [52,84]. Ammonia is a common environmental stressor in aquaculture, causing oxidative damage, immune suppression, and increased mortality in fish [85]. In our study, dietary RO supplementation markedly attenuated cumulative mortality in largemouth bass following exposure to acute ammonia stress. To our knowledge, this is the first study showing that RO can enhance resistance to environmental stressors in aquatic animals. Comparable protective effects have been documented with other dietary interventions. Existing literature demonstrates that dietary berberine supplementation in HF diets reduces mortality in blunt snout bream (*Megalobrama amblycephala*) post-ammonia challenge, while sodium acetate supplementation enhances ammonia tolerance in juvenile yellow catfish [16,84]. Both interventions enhanced survival by strengthening antioxidant capacity and immune responses. Probiotic supplementation has also been reported to improve ammonia resistance by modulating intestinal microbiota composition and promoting the production of SCFAs, which contribute to improved redox balance and mucosal immunity in fish [86,87,88]. In comparison, RO exhibits a broader spectrum of regulatory actions. In addition to activating the Nrf2 pathway and enhancing hepatic antioxidant enzyme activities, RO simultaneously modulated lipid metabolism, suppressed ER stress, and reshaped the gut microbiota toward SCFA-producing taxa such as *Faecalibaculum* and *Dubosiella*. Given that ammonia toxicity is tightly linked to ROS overproduction and oxidative damage, activation of Nrf2-mediated antioxidant defenses likely represents a key mechanism by which RO alleviates ammonia-induced mortality. Moreover, the RO-induced microbial shifts may indirectly enhance intestinal barrier integrity and nitrogen metabolism, further contributing to stress resistance. Overall, compared with other feed additives, RO provides multi-target protection by enhancing antioxidant defenses, regulating metabolism, and modulating gut microbiota, thus highlighting its potential as a promising additive to improve stress tolerance in fish.

### 4.6. Strengths and Limitations

This study provides the first evidence that dietary RO mitigates HF diet-induced metabolic and oxidative dysfunctions in a carnivorous aquaculture species, improving lipid metabolism, antioxidant capacity, gut microbiota composition, and ammonia stress tolerance.

However, several limitations should be noted: the study was conducted on a single species at the juvenile stage, with a short-term (8-week) feeding trial and only a single RO dose, leaving long-term safety, dose-response, and metabolomic validation unaddressed. The tissue-specific mechanism of PPARγ activation also remains unclear, and effects on product quality were not evaluated.

Future research should explore RO effects in other carnivorous species, longer-term farm-like trials, dose optimization and safety, interactions with other dietary interventions, and impacts on product quality. Addressing these points will clarify the practical applicability and underlying mechanisms of RO as a functional feed additive in aquaculture.

## 5. Conclusions

Dietary rosiglitazone (RO; 10 mg·kg^−1^) effectively alleviated HF diet-induced metabolic disorders and health impairments in juvenile largemouth bass. The high-fat diet significantly reduced growth performance, whereas RO supplementation improved the related parameters to levels comparable to those of the control group. Mechanistically, RO restored lipid homeostasis, enhanced antioxidant capacity through activation of the Nrf2 signaling pathway, and reduced lipid peroxidation, ER stress, and inflammatory responses. In addition, RO modulated gut microbiota composition by enriching SCFA-producing taxa, which may further contribute to improved metabolic balance and resilience under stress conditions. Collectively, these multi-target effects support the potential of RO as a promising functional feed additive for enhancing both health and production performance in carnivorous fish fed high-fat diets. Further studies should investigate tissue-specific PPARγ activation, dose optimization, and long-term safety under field conditions before practical application.

## Figures and Tables

**Table 1:** Formulation and nutrition composition of experimental diets.

Items	Con	HF	RO
Feed ingredients (%, dry matter)			
Fishmeal	50.00	50.00	50.00
Soy protein concentrate	13.00	13.00	13.00
Soybean meal	10.00	10.00	10.00
Wheat starch	7.00	7.00	7.00
Fish oil	3.00	7.00	7.00
Soybean oil	3.00	7.00	7.00
Vitamin premix ^1^	0.75	0.75	0.75
Mineral premix ^2^	0.75	0.75	0.75
Vitamin C	0.20	0.20	0.20
Monocalcium phosphate	1.50	1.50	1.50
Sodium carboxymethyl cellulose	1.00	1.00	1.00
Microcrystalline cellulose	9.80	1.80	1.799
Rosiglitazone ^3^	0.00	0.00	0.001
Total	100.00	100.00	100.00
Nutrition analysis, % dry matter basis premix ^4^			
Moisture	3.19	3.25	3.28
Crude protein	45.99	45.57	45.98
Crude lipid	10.24	18.08	18.25
Ash	12.18	12.13	12.04

^1^ Mixed vitamin (g/kg): Inositol 25 g, Cholin 100 g, Vitamin B_12_ 5 g, Niacin 25 g, Vitamin B_6_ 5 g, Folic acid 1 g, Vitamin B_2_ 5 g, Biotin 0.25 g, Vitamin B_1_ 5 g, Vitamin C 10 g, Vitamin K_3_ 1 g, Vitamin A 0.15 g, Vitamin E 2.5 g, Pantothenic acid 10 g, Vitamin D_3_ 0.00125 g. ^2^ Mixed minerals (g/kg): NaSeO_3_ 0.02 g, MgSO_4_·7H_2_O 147.4 g, (NH_4_)_2_MoS_4_ 0.06 g, CoCl_2_·6H_2_O 0.08 g, NaCl 49.8 g, KI 0.16 g, C_12_H_22_FeO_14_ 10.9 g, CuSO_4_·5H_2_O 0.62 g, ZnSO_4_·7H_2_O 4.67 g, MnSO_4_·H_2_O 3.12 g. ^3^ Rosiglitazone was purchased from Sinopharm Chemical Reagent Co., Ltd. (Beijing, China; website: http://en.reagent.com.cn/) with a purity of 99% (CAS No. 122320-73-4; Catalog No. XW0112232073401). ^4^ All were measured values.

**Table 2:** Sequences of primers used for quantitative real-time PCR.

Genes	Sequences (5′→3′)	Product Length (bp)	Primer Efficiency (%)	Accession Number
Antioxidant genes				
*nrf2*	F: CAGACGGGGAAACAAACAATG	170	101.3	XM_038720536.1
	R: GGGGTAAAATACGCCACAATAAC			
*keap1*	F: TCATTGGGGAATCACATCTTTG	199	97.4	XM_038713666.1
	R: TGTCCAGAAAAGTGTTGCCATC			
*sod*	F: TCCCCACAACAAGAATCATGC	180	96.5	XM_038708943.1
	R: TCATCAGCCTTCTCGTGGA			
*cat*	F: CTGCTGTTCCCGTCCTTCAT	154	102.4	XM_038704976.1
	R: GGTAGCCATCAGGCAAACCT			
*gpx*	F: GCAATCAGTTTGGACATCAGG	126	100.1	XM_038697220.1
	R: TTCCATTCACATCCACCTTCT			
*gst*	F: AATGGAGCACAAGTCACAGGA	107	100.7	XM_038724634.1
	R: ACAAGCAGGCAGCATAGGA			
Inflammation genes				
*il-10*	F: CGGCACAGAAATCCCAGAGC	119	103.8	XM_038723321.1
	R: CAGCAGGCTCACAAAATAAACATCT			
*tnfα*	F: TCCAGCATCACACGGAAGAAGT	129	101.9	XM_038729256.1
	R: CAGCAGATGTCAGAGCCTCAGT			
*il-1β*	F: GATGCTCTTTAACTCCTCCT	88	99.9	XM_038733429.1
	R: CACCAACTTGTACATGTCCT			
*nf-κb*	F: CCACTCAGGTGTTGGAGCTT	127	98.4	XM_038699793.1
	R: TCCAGAGCACGACACACTTC			
Peptide transporters				
*grp78*	F: TTGCCGATGACGACGAAA	180	104.6	XM_038733280.1
	R: CAATCAGACGCTCACCCT			
*eif2α*	F: CCTCGTTTGTCCGTCTGTATC	92	98.6	XM_038693619.1
	R: GCTGACTCTGTCGGCCTTG			
*chop*	F: GATGAGCAGCCTAAGCCACG	153	101.7	XM_038701049.1
	R: AACAGGTCAGCCAAGAAGTCG			
*atf6*	F: GCAACACCTGGACGACAACCT	111	99.2	XM_038724893.1
	R: GGCTCTGCTTTCACCTGGAACA			
*ire1*	F: CTTGTGTCGAGTGGCGATGGT	187	100.6	XM_038712961.1
	R: CGTTGGCAGAGGAGAAGGTGAG			
Amino acid transporters				
*srebp1*	F: AGTCTGAGCTACAGCGACAAGG	127	99.3	XM_038699585.1
	R: TCATCACCAACAGGAGGTCACA			
*dgat1*	F: AGACTGGTGGAACTCTGAGAC	171	105.8	XM_038724648.1
	R: ACTAGGTACTCGTGGAAGAAGG			
*pparγ*	F: ATGTCACACAACGCCATTCG	135	100.8	XM_038695875.1
	R: GTACAGATGCCGGGACAGAG			
*acox1*	F: CAGTTCTGTTCGTCACCAGTC	173	97.5	XM_038695271.1
	R: CGTTGATGTCTCCGCTGATG			
*cpt1*	F: AGCCCCACCCCAACCTACCAG	283	96.3	XM_038705335.1
	R: CGGCCCTCACGGAATAAACGC			
*hsl*	F: GAAGATCATATCCAGCGGCATC	157	104.9	XM_038725627.1
	R: TCCATAGGCATTGAGGCACTT			
Housekeeping genes				
*β-actin*	F: TTTATGGATAGAGCCGGGCA	161	102.5	XM_038695351.1
	R: CTTCCATGGCTGAACTTTGGG			
*ef1α*	F: TGCTGCTGGTGTTGGTGAGTT	147	103.2	XM_038724777.1
	R: TTCTGGCTGTAAGGGGGCTC			

Note: *nrf2* = nuclear factor erythroid 2-related factor 2; *keap1* = kelch-like ECH-associated protein 1; *sod* = superoxide dismutase; *cat* = catalase; *gpx* = glutathione peroxidase; *gst* = glutathione s-transferase; *il-10* = interleukin-10; *tnfα* = tumor necrosis factor α; *il-1β* = interleukin 1β; *nf-κb* = nuclear factor kappa-B; *grp78* = glucose regulated protein 78; *eif2α* = eukaryotic initiation factor-2α; *chop* = C/EBP-homologous protein; *atf6* = activating transcription factor 6; *ire1* = inositol-requiring enzyme 1; *srebp1* = sterol regulatory element binding protein 1; *dgat1* = diacylglycerol oacyltransferase 1; *pparγ* = peroxisome proliferator-activated receptor γ; *acox1* = acyl-CoA oxidase 1; *cpt1* = carnitine palmitoyltransferase1; *hsl* = hormone-sensitive lipase; *β-actin* = beta-actin; *ef1α* = elongation factor 1α.

**Table 3:** Growth performance of largemouth bass under dietary treatment.

Items	Con	HF	RO	*p*
Growth and feed performance (*n* = 3)				
SR ^1^, %	97.78 ± 1.11	98.89 ± 1.11	98.89 ± 1.11	0.729
IBW, g/fish	3.29 ± 0.02	3.21 ± 0.03	3.30 ± 0.03	0.146
FBW, g/fish	39.10 ± 0.85 ^b^	35.47 ± 0.24 ^a^	36.60 ± 0.79 ^ab^	0.024
FI ^2^, g/fish	38.04 ± 0.75 ^b^	34.71 ± 0.24 ^a^	34.68 ± 0.28 ^a^	0.004
FR ^3^, %/day	3.52 ± 0.02	3.52 ± 0.03	3.41 ± 0.10	0.636
WGR ^4^, %	1088.45 ± 18.03 ^b^	1004.86 ± 14.80 ^a^	1009.01 ± 21.16 ^a^	0.029
SGR ^5^, %/day	4.85 ± 0.03 ^b^	4.71 ± 0.03 ^a^	4.72 ± 0.04 ^a^	0.029
FCR ^6^	1.04 ± 0.01	1.07 ± 0.01	1.03 ± 0.03	0.319
PER ^7^, %	204.61 ± 1.43	203.99 ± 1.87	212.74 ± 6.68	0.430
Body indexes and intestinal indicators (*n* = 9)				
HSI ^8^, %	0.95 ± 0.10 ^a^	1.32 ± 0.12 ^b^	1.28 ± 0.10 ^ab^	0.047
VSI ^9^, %	8.96 ± 0.24 ^a^	10.54 ± 0.39 ^b^	9.02 ± 0.27 ^a^	0.001
IPF ^10^, %	1.80 ± 0.17 ^a^	2.87 ± 0.19 ^b^	2.28 ± 0.16 ^ab^	<0.001
CF ^11^, %	2.69 ± 0.04	3.17 ± 0.31	2.46 ± 0.25	0.155

Note: IBW = initial body weight; FBW = final body weight; WGR = weight gain rate; SGR = specific growth rate; FCR = feed conversion rate; PER = protein efficiency ratio; FI = apparent feed intake; FR = feeding rate; SR = survival rate; VSI = viscerosomatic index; HSI = hepatosomatic index; CF = condition factor; IPF = intraperitoneal fat ratio. All data presented as means ± SEM (*n* = 3 for growth and feed performance; *n* = 9 for body indexes and intestinal indicators). Means of the same parameter with different superscripts are significantly different (*p* < 0.05). ^1^ SR = 100 × (final fish number/initial fish number); ^2^ FI = total feed supplied − uneaten feed collected by siphoning; ^3^ FR = 100 × FI/[(W_f_ + W_d_ + W_i_)/2]/days, where W_f_ is the final total weight, W_d_ is the total weight of dead fish, and W_i_ is the initial total weight. ^4^ WGR = 100 × (W_f_ + W_d_ − W_i_)/W_i_. ^5^ SGR = 100 × [ln (FBW/IBW)]/days. ^6^ FCR = FI/(FBW − IBW). ^7^ PER = (W_f_ − W_i_)/(FI × CP_feed_), where CP_feed_ is the feed protein content. ^8^ HSI = 100 × (liver weight)/(body weight). ^9^ VSI = 100 × (viscera weight)/(body weight). ^10^ IPF = 100 × (Intraperitoneal fat weight/body weight). ^11^ CF = body weight/body length^3^.

**Table 4:** Main gut microbial taxa altered by dietary treatments.

Taxonomic Level	Increased Taxa (vs. Con)	Decreased Taxa (vs. Con)
HF group	Firmicutes, Mycoplasma	Proteobacteria, Plesiomonas
RO group	Firmicutes, Actinobacteriota, *Mycoplasma*, *Microbacteriaceae_unclassified*, *Dubosiella*, *Faecalibaculum*	Fusobacteriota, Proteobacteria, *Cetobacterium*, *Plesiomonas*

## Data Availability

Data will be made available on request.
